# Annotations

**Published:** 1887-10-15

**Authors:** 


### ANNOTATIONS.

**New Wars: New Weapons.** MEDICAL men must look to their scientific and intellectual armamentaria. There are many significant indications that "apprenticeship science" and "rule of thumb practice" will not satisfy the reading patient of to-day. The *Century Magazine* is now publishing a series of popular but exact articles on the "Digestibility of Food," and similar physiological topics. Thirty years ago no magazine for the general public would have admitted such papers, for the simple reason that they would not have been acceptable to its readers. Now, however, it is evident that there is a demand for pabulum of the scientifico-medical kind; and it may be relied upon that enterprising publishers in America and other countries will not leave the demand unsatisfied. Practitioners should realise that they are working out some of the principles of Darwinism in their own persons. The man who hates books and prides himself on being "purely practical," cannot impose upon a reading public as he has done upon a public which did not read. His game is played out. It should not be the least difficult even for the busiest general practitioner to keep himself well ahead of the most industrious dabbler in medical science among his patients. Because, however lazy the doctor may have been as a student, he cannot but have acquired a knowledge of principles and details far in excess of that possessed by any mere layman; and with his patients so handicapped at the outset, a very little regular and intelligent reading on his part should leave them well in the rear. But that is the fence so many hardworked family practitioners "boggle" at. They will not do the regular reading. The leading medical journals contain an almost infinite variety of scientific, practical, curious, and interesting matter; and if there be no time for more special work, a steady and thoughtful perusal of their contents from week to week will keep a man fairly abreast of the practical progress of his time.

**The Digestibility of Food.** As a sample of what is being provided by the managers of *The Century* for its readers, we give one or two extracts from an article in the September number on this topic:—"The ways in which the experiments to test the digestibility of foods are made very ingenious and interesting. Physiologists use the salivary glands or stomach or intestine of a living animal much as chemists do their bottles and retorts and test-tubes. One easily gets into the way of regarding an animal as simply an organism manifesting certain reactions under given conditions, and in not a few European laboratories a janitor is readily induced by the price of a few months' supply of beer, or a student by his scientific ardour, to take this same altruistic view of his own physical organism." In one of a series of experiments conducted in the physiological laboratory at Munich by Dr. Rubner, the subject, a healthy Bavarian labourer, lived for three days on bread and water, a diet the monotony of which was more endurable than one of meat, fish, or almost any other single substance. The man was able to eat 1,185 grammes (about 2 lb. 10 oz.) per day. This contained 670 grammes of carbohydrates, 81 of protein, and some fatty matter. Of the 670 grammes of carbohydrates, 665, or about 99 per cent., were digested; and of the 81 grammes of protein, 68, or 87 per cent. The quantity of fat in the bread was too small to admit of an accurate test of its digestibility. In another series of experiments in the same laboratory the digestibility of meat, fish, etc., was similarly tested. This time the subject was a medical student. He was not able to consume so much as two pounds of meat per day, although he had no other food of any kind, and the dishes were cooked in every variety of way to suit his taste. On the second day he took his food with great difficulty; and on the third he could hardly swallow it at all. The result will surprise a good many people who have no familiarity with the subject. No less than 99 per cent. of the protein of the meat and fish were completely digested. "Other trials with meat and fish brought similar results, and it is reasonably safe to say that when a healthy person, with sound digestive organs, eats ordinary meat or fish in proper quantity, all or nearly all of the protein is digested."

**Butter or Butterine.** In America, as in this country, there have been fierce legislative and other fights about butter and butterine, or oleomargarine, as some prefer it to be called. One result of the quarrels has been the bringing to light of the exact chemical constitution of both. "In chemical composition oleomargarine stands between meat-fat and butter." It is made from beef-fat and lard or pig's-fat. It is therefore, if pure, a perfectly wholesome substance. In the manufacture, part of the stearine, the least digestible constituent of fat, is removed. A little butter, and sometimes oil, such as cotton-seed oil, is added to give it flavour. People who desire to possess exact knowledge should remember that all these fatty substances—meat-fat, butter, and oil—are very similar in ultimate composition. There are two important differences—not important as affecting their wholesomeness, but as affecting their digestibility and flavour. The meat-fat contains more of the indigestible stearine; and the butter has substances peculiar to itself, as butyrin, caproin, etc., to which it owes its special flavour. Much has been written about the relative digestibility of butter and oleomargarine. There are certain comparative tests on record made by Professor Mager in Holland. "In these from 97.7 to 98.4 per cent. of the fat of the butter, and from 96.1 to 96.3 of the fat of the oleomargarine were digested. The average difference was 1.6 per cent. in favour of the butter." With regard, therefore, to this question of relative digestibility, "the facts at hand and the general impression of special students of the subjects . . . are to the effect that probably, for healthy persons, the difference between butter and oleomargarine in ease and completeness of digestion would be at most very slight, but that for people with enfeebled digestion and for infants, butter may perhaps . . . have the advantage." So much for the pronouncements of exact science, American and German. But everyone should understand that though the stomach is in some senses a chemical laboratory, yet it differs from other laboratories in possessing a nervous system, and vitality (whatever that may be), and distinct idiosyncrasies of its own. We confess also to a certain sympathy with those foods which are elaborated in Nature's workshops as against those manufactured by "art and man's device." When Nature fashioned the cow for the production of cream she never thought of dividends. We must always keep a sharp eye on the man who has an "axe to grind."

